# Drivers of international variation in prevalence of disabling low back pain: Findings from the Cultural and Psychosocial Influences on Disability study

**DOI:** 10.1002/ejp.1255

**Published:** 2018-06-27

**Authors:** D. Coggon, G. Ntani, K.T. Palmer, V.E. Felli, F. Harari, L.A. Quintana, S.A. Felknor, M. Rojas, A. Cattrell, S. Vargas‐Prada, M. Bonzini, E. Solidaki, E. Merisalu, R.R. Habib, F. Sadeghian, M.M. Kadir, S.S.P. Warnakulasuriya, K. Matsudaira, B. Nyantumbu‐Mkhize, H.L. Kelsall, H. Harcombe

**Affiliations:** ^1^ Medical Research Council Lifecourse Epidemiology Unit University of Southampton UK; ^2^ Arthritis Research UK/MRC Centre for Musculoskeletal Health and Work University of Southampton UK; ^3^ School of Nursing University of São Paulo Brazil; ^4^ Corporación para el Desarrollo de la Producción y el Medio Ambiente Laboral – IFA (Institute for the Development of Production and the Work Environment) Quito Ecuador; ^5^ Department of Industrial Engineering School of Engineering Pontificia Universidad Javeriana Bogotá Colombia; ^6^ Southwest Center for Occupational and Environmental Health The University of Texas Health Science Center at Houston School of Public Health TX USA; ^7^ Center for Disease Control and Prevention/National Institute for Occupational Safety and Health Atlanta GA USA; ^8^ Program Health, Work and Environment in Central America Institute for Studies on Toxic Substances (IRET) National University of Costa Rica Heredia Costa Rica; ^9^ North East London NHS Foundation Trust Goodmayes Hospital Ilford Essex UK; ^10^ Center for Research in Occupational Health (CiSAL) Universitat Pompeu Fabra Barcelona Spain; ^11^ CIBER of Epidemiology and Public Health Barcelona Spain; ^12^ IMIM (Hospital del Mar Research Institute) Barcelona Spain; ^13^ Unidad Central de Contingencias Comunes (U3C) Mutua Asepeyo Spain; ^14^ Department of Clinical Sciences and Community Health Università degli Studi di Milano Milan Italy; ^15^ Department of Social Medicine Medical School University of Crete Heraklion Greece; ^16^ Institute of Technology Estonian University of Life Sciences Tartu Estonia; ^17^ Department of Environmental Health Faculty of Health Sciences American University of Beirut Lebanon; ^18^ Department of Occupational Health School of Public Health Shahroud University of Medical Sciences Shahroud Iran; ^19^ Department of Community Health Sciences Aga Khan University Karachi Pakistan; ^20^ Department of Allied Health Sciences Faculty of Medical Sciences University of Sri Jayawardenepura Gangodawila, Nugegoda Sri Lanka; ^21^ Department for Medical Research and Management for Musculoskeletal Pain 22nd Century Medical and Research Center Faculty of Medicine The University of Tokyo Hospital Tokyo Japan; ^22^ National Institute for Occupational Health National Health Laboratory Service Johannesburg South Africa; ^23^ Faculty of Health Sciences School of Public Health University of Witwatersrand Johannesburg South Africa; ^24^ Department of Epidemiology and Preventive Medicine School of Public Health and Preventive Medicine Monash University Melbourne Vic. Australia; ^25^ Department of Preventive and Social Medicine University of Otago Dunedin New Zealand

## Abstract

**Background:**

Wide international variation in the prevalence of disabling low back pain (LBP) among working populations is not explained by known risk factors. It would be useful to know whether the drivers of this variation are specific to the spine or factors that predispose to musculoskeletal pain more generally.

**Methods:**

Baseline information about musculoskeletal pain and risk factors was elicited from 11 710 participants aged 20–59 years, who were sampled from 45 occupational groups in 18 countries. Wider propensity to pain was characterized by the number of anatomical sites outside the low back that had been painful in the 12 months before baseline (‘pain propensity index’). After a mean interval of 14 months, 9055 participants (77.3%) provided follow‐up data on disabling LBP in the past month. Baseline risk factors for disabling LBP at follow‐up were assessed by random intercept Poisson regression.

**Results:**

After allowance for other known and suspected risk factors, pain propensity showed the strongest association with disabling LBP (prevalence rate ratios up to 2.6, 95% CI: 2.2–3.1; population attributable fraction 39.8%). Across the 45 occupational groups, the prevalence of disabling LBP varied sevenfold (much more than within‐country differences between nurses and office workers), and correlated with mean pain propensity index (*r* = 0.58).

**Conclusions:**

Within our study, major international variation in the prevalence of disabling LBP appeared to be driven largely by factors predisposing to musculoskeletal pain at multiple anatomical sites rather than by risk factors specific to the spine.

**Significance:**

Our findings indicate that differences in general propensity to musculoskeletal pain are a major driver of large international variation in the prevalence of disabling low back pain among people of working age.

## 1 Introduction

Low back pain (LBP) is the leading cause of disability globally (Hoy et al., 2014), and a major contributor to incapacity for work among young and middle‐aged adults (Bevan et al., 2009). Risk factors for its incidence and/or persistence include activities such as heavy lifting that load the spine mechanically (Lötters et al., 2003), tendency to somatize (Pincus et al., 2002; Vargas‐Prada et al., 2013), low mood (Pincus et al., 2002; Ramond et al., 2011; Vargas‐Prada et al., 2013), psychosocial stressors in the workplace (Lang et al., 2012) and adverse beliefs about the prognosis of back disorders (Ramond et al., 2011). In Europe, the consistency of its association with mechanical loading has prompted legislation requiring employers to control manual handling in the workplace through appropriate design of tasks and equipment (European Agency for Safety and Health at Work, 1990). However, in randomized controlled trials, reductions in LBP from ergonomic interventions have been minimal (Driessen et al., 2010; Verbeek et al., 2012). Moreover, the descriptive epidemiology of LBP suggests that there are other more important determinants. For example, in Britain there was an eightfold increase in long‐term incapacity for work because of LBP between 1950 and the early 1990s – a change too large to be explained by known causes (Clinical Standards Advisory Group, 1994).

Given the established role of psychological factors in the occurrence of LBP, we hypothesized that trends in disability from back disorders could be a consequence of changes in health beliefs and expectations, and that culturally determined differences in health beliefs might lead also to large international variation in prevalence (Coggon, 2005). To test this theory, we initiated the CUPID (Cultural and Psychosocial Influences on Disability) study, in which information about musculoskeletal pain, associated disability and potential risk factors was collected from workers sampled from 47 occupational groups across 18 countries (Coggon et al., 2012). Analysis of cross‐sectional data at baseline confirmed that there were up to sevenfold differences between occupational groups in the 1‐month prevalence of disabling LBP, but the variation was not explained either by established risk factors or by knowledge and beliefs about LBP (Coggon et al., 2013a). It did, however, correlate with differences across occupational groups in the prevalence of disabling wrist/hand pain, suggesting that the two complaints might be driven importantly by one or more shared risk factors that are associated with a general propensity to experience and report musculoskeletal pain and associated disability (Coggon et al., 2013a). The existence of such propensity would accord with the observation that musculoskeletal pain often affects multiple anatomical sites, either simultaneously or closely in time (Natvig et al., 2001; IJzelenberg and Burdorf, 2004; Haukka et al., 2006; Coggon et al., 2013b), and that pain elsewhere predicts the future occurrence of LBP (Papageorgiou et al., 1996; Smith et al., 2004).

To explore the extent to which differences in general propensity to musculoskeletal pain might account for variation in the prevalence of LBP between occupational groups, we analysed longitudinal data from the CUPID study, looking at baseline risk factors for the 1‐month prevalence of disabling LBP at follow‐up, and taking as an index of pain propensity, the number of anatomical sites other than the low back that were reported as painful in the 12 months before baseline. We opted for a longitudinal design rather than a cross‐sectional analysis because it would avoid bias from simultaneous reporting of risk factors and outcomes.

## 2 Methods

The methods of the CUPID study have been reported in detail elsewhere (Coggon et al., 2012). Ethical approval for the investigation was provided by relevant ethics committees in each of the 18 participating countries.

### 2.1 Study sample

The 47 occupational groups that made up the initial study sample fell into three broad categories – nurses, office workers and ‘other workers’ mainly carrying out repetitive manual tasks. During 2006–11, men and women aged 20–59 years who were eligible for inclusion according to pre‐specified criteria, were identified (in most occupational groups from employers’ records) and invited to complete a baseline questionnaire, either by self‐administration, or in some occupational groups, at interview (overall response rate 70%).

### 2.2 Baseline questionnaire and specification of personal risk factors

The questionnaire was originally drafted in English, and then translated to local languages where necessary, with checks for accuracy by independent back‐translation. Among other things, it covered: sex; age; smoking habits (never smoked, ex‐smoker or current smoker); hours worked per week (< or ≥50 h per week); other psychosocial aspects of work, (incentives from piecework or bonuses; time pressure; lack of choice in what work was done, how and when; lack of support from colleagues or supervisor/manager; job dissatisfaction; and perceived job insecurity if off work for 3 months with illness); occupational lifting (whether an average working day entailed lifting weights ≥25 kg by hand); mental health; somatizing tendency; adverse beliefs about LBP (work‐relatedness, prognosis and effects of physical activity); and recent experience of musculoskeletal pain.

Mental health was assessed through questions taken from the Short Form‐36 (SF‐36) questionnaire (Ware and Sherbourne, 1992), and was graded to three levels (good, intermediate and poor) corresponding to approximate thirds of the distribution of scores in the full study sample. Somatizing tendency was determined through questions taken from the Brief Symptom Inventory (Derogatis and Melisoratos, 1983), and was graded according to the number of somatic symptoms from a total of five (faintness or dizziness, pains in the heart or chest, nausea or upset stomach, trouble getting breath, hot or cold spells) that had been at least moderately distressing during the past week. Questions on adverse beliefs were adapted from the Fear Avoidance Beliefs Questionnaire (Waddell et al., 1993). Participants were classed as having adverse believes about work‐relatedness if they completely agreed that back pain is commonly caused by work; about its relationship to physical activity if they completely agreed that for someone with back pain, physical activity should be avoided as it might cause harm, and that rest is needed to get better; and about its prognosis if they completely agreed that neglecting such problems can cause serious harm, and completely disagreed that such problems usually get better within 3 months.

The questions about musculoskeletal pain focused on 10 anatomical sites (low back; neck; and right and left shoulder, elbow, wrist/hand and knee), which were illustrated diagrammatically. For each site, participants were asked whether they had experienced pain during the past 12 months that had lasted for longer than a day. In addition, those who reported LBP were asked whether it had been present during the past month, and if so, whether during that time it had made it difficult or impossible to get dressed, do normal jobs around the house or cut toe nails (which we classed as disabling LBP).

### 2.3 Group‐level risk factors

In addition to the information obtained from questionnaires, the lead investigator in each country provided baseline information about group‐level factors (variables which took an identical value for all members of the same occupational group) that might impact on disability from musculoskeletal symptoms. These included: the unemployment rate in the community from which the occupational group came, whether it was necessary to pay for primary medical care, and the availability of: pay during sickness absence, financial support for ill‐health retirement, social security for long‐term unemployment and compensation for work‐related LBP.

### 2.4 Follow‐up

After an interval of approximately 14 months, participants in all but two of the occupational groups (manual workers in Costa Rica and office workers in South Africa) were asked to answer a shorter follow‐up questionnaire – as before by self‐administration or at interview. This included questions about experience of LBP for a day or longer in the past month, and again asked whether that pain had made it difficult or impossible to get dressed, do normal jobs around the house or cut toe nails (disabling LBP).

### 2.5 Statistical analysis

Statistical analysis was carried out with Stata v.12.1 software (Stata Corp LP 2012, Stata Statistical Software: Release 12.1; College Station TX, USA). For each participant, we derived a ‘pain propensity index’ defined by the number of anatomical sites other than the low back that were reported as having been painful in the 12 months before baseline. We used simple descriptive statistics to explore the relationship of this index to other personal characteristics at baseline.

We then applied Poisson regression (with robust standard errors) to examine the association of disabling LBP in the past month at follow‐up as an outcome variable with potential risk factors at baseline. To allow for possible clustering by occupational group, we used random intercept models. Associations were summarized by prevalence rate ratios (PRRs) with 95% confidence intervals (CIs). We first fitted a model that included personal risk factors, including pain propensity index.

Next, we explored the role of group‐level risk factors, analysing each in a separate Poisson regression model that also included all of the personal risk factors. As well as the group‐level measures that had been provided by local investigators, we also examined five group‐level variables that were derived from the individual questionnaires (the mean pain propensity index in the occupational group, and the group prevalence of knowing someone outside work with low back pain, and of adverse beliefs about LBP regarding its work‐relatedness, prognosis and the effects of physical activity). These were included to address the original hypothesis of the CUPID study that differences between occupational groups in the prevalence of musculoskeletal pain and associated disability might be importantly determined by differences in culturally determined health beliefs and expectations. Thus, for example, the group prevalence of knowing someone outside work with LBP was an indicator of the prominence of LBP as a symptom in the participant's community, which might influence how an individual perceived and responded to back symptoms when they occurred.

We then fitted a single model incorporating all of the personal and group‐level factors that had shown significant (*p* < 0.05) associations with disabling LBP in the earlier analyses, and from the PRRs obtained, we estimated population attributable fractions (PAFs) for each factor. These indicated the proportion of cases in the study population that would be eliminated if (after adjustment for other risk factors), the prevalence of disabling LBP in those with exposure to the risk factor were reduced to that in those unexposed. While they do not necessarily assume or imply that the risk factor caused disabling LBP to develop, persist or recur, they illustrate its potential importance as a driver of the prevalence of the symptom. Confidence intervals for PAFs were calculated by bootstrapping with 250 repetitions per estimate (Efron, 1979).

To explore the extent to which pain propensity and other risk factors might explain variation between occupational groups in the prevalence of disabling LBP, we compared the numbers of cases by occupational group with the numbers that would have been expected: (1) based only on the overall prevalence of disabling LBP in the full study sample; (2) calculated from a Poisson regression model that adjusted for pain propensity index (using predicted probabilities generated by Stata); and (3) calculated from the final Poisson regression model that included all statistically significant risk factors. The extent of variation was characterized by the geometric standard deviation of the ratios of observed to expected numbers. To set the results in context, we used random simulations to explore the expected distributions of geometric standard deviations under the assumption that each individual's probability of disabling LBP was that which would have been predicted from the relevant Poisson regression model given his/her exposure to risk factors. Thus, for the first and simplest analysis, the simulations assumed that each person had a probability of disabling LBP equal to the overall prevalence; for the second analysis, the simulations assumed that a person's probability of disabling LBP was that which would be expected from their pain propensity index, taking no other information into account; while the simulations for the third analysis, assumed that each person's probability of LBP was that predicted from the final Poisson regression model.

## 3 Results

Within the 45 occupational groups that contributed to the longitudinal component of the study, 11 992 participants answered the baseline questionnaire, including 11 710 who provided complete information about pain at anatomical sites other than the low back during the 12 months before baseline. Among the latter, 9055 (3083 men) answered the items about LBP in the follow‐up questionnaire, giving a usable response rate of 77.3%. The number of responders by occupational group ranged from 39 to 633, with follow‐up rates >70% in 36 of the 45 groups. Follow‐up was marginally higher in older participants, those with greater pain propensity, and those with disabling LBP in the month before baseline (Table 1).

**Table 1:** Response rates at follow‐up according to demographic characteristics and report of pain at baseline

Baseline characteristic	Number of participants who provided adequate information at baseline	Number (%) with usable follow‐up
Sex		
Male	4065	3083 (75.8%)
Female	7645	5972 (78.1%)
Age (years)		
20–29	2817	2087 (74.1%)
30–39	3784	2913 (77.0%)
40–49	3275	2602 (79.5%)
50–59	1834	1453 (79.2%)
Pain propensity score		
0	3598	2690 (74.8%)
1	2582	1972 (76.4%)
2	1973	1547 (78.4%)
3	1522	1186 (77.9%)
4	846	699 (82.6%)
5	591	482 (81.6%)
6	278	218 (78.4%)
7	194	158 (81.4%)
8	62	52 (83.9%)
9	64	51 (79.7%)
Disabling LBP in past month^a^		
No	9046	7002 (77.4%)
Yes	2529	1995 (78.9%)

a Data on disabling LBP in the past month at baseline were missing for 135 participants.

Among the 9055 participants who were suitable for analysis, the pain propensity index at baseline varied from 0 (2690 participants) to 9 (51 participants), with mean 1.9, median 1 and interquartile range 0–3. Mean values were higher in women than men, at older ages, in participants with poor mental health, and in those who reported distress from common somatic symptoms (Table 2). However, there was little difference in relation to smoking habits.

**Table 2:** Relationship of pain propensity index to personal characteristics

Characteristic	Pain propensity index^a^		
	Mean (95% CI)	Median	Inter‐quartile range
Sex			
Male	1.4 (1.3, 1.4)	1	0–2
Female	2.2 (2.1, 2.2)	2	1–3
Age (years)			
20–29	1.5 (1.4, 1.5)	1	0–2
30–39	1.8 (1.7, 1.8)	1	0–3
40–49	2.1 (2.0, 2.2)	2	0–3
50–59	2.4 (2.3, 2.5)	2	1–4
Smoking habits			
Never smoked	1.9 (1.8, 1.9)	1	0–3
Ex‐smoker	2.0 (1.9, 2.1)	2	0–3
Current smoker	1.8 (1.7, 1.9)	1	0–3
Mental health			
Good	1.6 (1.5, 1.7)	1	0–2
Intermediate	1.9 (1.9, 2.0)	1	0–3
Poor	2.2 (2.2, 2.3)	2	0–3
Somatising tendency (number of distressing somatic symptoms in past week)			
0	1.4 (1.4, 1.5)	1	0–2
1	2.2 (2.1, 2.3)	2	1–3
≥2	3.0 (2.9, 3.2)	3	1–5

a For definition of pain propensity index see text.

At follow‐up, 2003 participants (22%) reported disabling LBP in the past month, including 1663 (83%) who had also reported LBP in the 12 months before baseline, and 1027 (51%) with disabling LBP in the month before baseline. Table 3 summarizes the association of disabling LBP at follow‐up with personal risk factors assessed at baseline. With adjustment for occupational group by random intercept modelling, risk was notably higher in women than men (PRR: 1.4, 95% CI: 1.2–1.5), at older ages (PRR: 1.4, 95% CI: 1.2–1.6, for age 50–59 years vs. 20–29 years) and in participants with greater tendency to somatize (PRR: 1.4, 95% CI: 1.3–1.6, for report of ≥2 vs. 0 distressing somatic symptoms). However, after allowance for these and other covariates, pain propensity was by far the strongest risk factor. PRRs relative to a pain propensity index of zero increased progressively from 1.4 (95% CI: 1.2–1.5) for a value of 1, to 2.6 (95% CI: 2.2–3.1) for values ≥6.

**Table 3:** Associations of disabling low back pain in past month at follow‐up with personal risk factors at baseline

Risk factor	Number of subjects	Number with disabling LBP in past month at follow‐up	Association with disabling low back pain
			Prevalence rate ratio (95% CI)^d^
Sex			
Male	3083	446	1
Female	5972	1557	1.4 (1.2, 1.5)^c^
Age (years)			
20–29	2087	365	1
30–39	2913	606	1.1 (1.0, 1.2)
40–49	2602	649	1.3 (1.1, 1.4)^c^
50–59	1453	383	1.4 (1.2, 1.6)^c^
Smoking status			
Never smoked	5850	1322	1
Ex‐smoker	1291	283	1.1 (1.0, 1.3)^a^
Current smoker	1892	394	1.1 (1.0, 1.2)
Missing	22	4	
Lifting weights ≥25 kg	3237	772	1.1 (1.0, 1.2)
Psychosocial aspects of work			
Work for >50 h per week	2039	343	1.0 (0.9, 1.1)
Time pressure at work	6754	1586	1.1 (1.0, 1.2)^a^
Incentives at work	2494	594	1.1 (1.0, 1.2)^a^
Lack of support at work	2341	604	1.1 (0.9, 1.2)
Job dissatisfaction	1759	395	1.0 (0.9, 1.1)
Lack of job control	1811	408	1.0 (0.9, 1.1)
Job insecurity	2665	658	1.1 (1.0, 1.2)^a^
Number of distressing somatic symptoms in past week			
0	5425	854	1
1	1973	529	1.3 (1.2, 1.5)^c^
2+	1605	607	1.4 (1.3, 1.6)^c^
Missing	52	13	
Mental health			
Good	3596	658	1
Intermediate	2735	573	1.1 (1.0, 1.2)
Poor	2690	766	1.3 (1.1, 1.4)^c^
Missing	34	6	
Adverse health beliefs about low back pain			
Work‐relatedness	3117	854	1.1 (1.0, 1.2)^b^
Physical activity	1726	401	1.0 (0.9, 1.1)
Prognosis	1262	332	1.1 (1.0, 1.3)^a^
Individual pain propensity index			
0	2690	301	1
1	1972	329	1.4 (1.2, 1.5)^c^
2	1547	347	1.7 (1.5, 1.9)^c^
3	1186	343	2.0 (1.8, 2.3)^c^
4	699	230	2.1 (1.8, 2.4)^c^
5	482	204	2.4 (2.0, 2.9)^c^
6+	479	249	2.6 (2.2, 3.1)^c^

a *p* < 0.05.

b *p* < 0.01.

c *p* < 0.001.

d Prevalence rate ratio with 95% confidence interval, derived from a single Poisson regression model that included all of the risk factors in the table.

Table 4 shows results from a series of regression models, each of which included a group‐level variable as well as the personal risk factors examined in Table 3. Only one of the group‐level risk factors was significantly associated with disabling LBP at follow‐up – lack of social security support for long‐term unemployment (PRR: 1.3, 95% CI: 1.0–1.6).

**Table 4:** Associations of disabling low back pain in past month at follow‐up with group‐level risk factors at baseline

Risk factor^b^	Number of occupational groups exposed	Level of exposure		Association with disabling low back pain in past month
		Mean	SD	Prevalence rate ratio (95% CI)
Group prevalence (%) of adverse beliefs about low back pain				
Work‐relatedness^c^	45	32.9	19.9	1.1 (0.9, 1.2)
Physical activity^c^	45	18.9	17.9	1.0 (0.9, 1.1)
Prognosis^c^	45	12.5	8.5	1.0 (0.9, 1.1)
Group prevalence (%) of knowing someone outside work with low back pain^c^	45	59.9	14.0	1.0 (0.9, 1.1)
Availability of full sick pay in first 3 months absence	24			0.9 (0.7, 1.1)
Availability of financial support for ill‐health retirement (sometimes or usually)	26			0.9 (0.7, 1.1)
Lack of social security for long‐term unemployment	19			1.3 (1.0, 1.6)^a^
Availability of compensation (any) for work‐related musculoskeletal disorders of back	36			0.9 (0.7, 1.2)
Unemployment rate ≥10%	11			1.3 (1.0, 1.7)
Payment for primary care (part or full)	18			1.1 (0.9, 1.4)
Group mean propensity index^c^	45	1.8	0.7	1.1 (0.9, 1.2)

a *p* < 0.05.

b Each risk factor was examined independently in a separate Poisson regression model with adjustment for all of the risk factors in Table 2.

c Risk estimates for continuous variables are for an increase of one standard deviation.

To assess the potential importance of pain propensity and other risk factors at a population level, we entered all that were statistically significant into a single Poisson regression model, and calculated PAFs from the PRRs that were estimated. The largest PAF was for individual pain propensity (39.8%, 95% CI: 34.0–45.7%, for values >0), followed by female sex (20.3%), older age (16.3% for ages 30–59 vs. 20–29 years) and somatizing tendency (15.1% for ≥1 vs. 0 distressing symptoms). For the combination of individual pain propensity and/or somatizing tendency (32.7% of the study sample), the PAF was 54.9% (95% CI: 47.5–62.3%).

**Figure 1:**
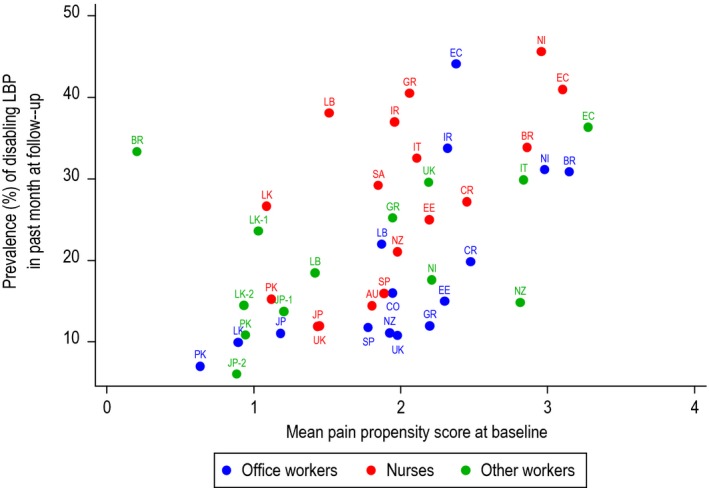
Prevalence of disabling low back pain and mean pain propensity score by occupational group. Key to countries: AU, Australia; BR, Brazil; CO, Colombia; CR, Costa Rica; EC, Ecuador; EE, Estonia; GR, Greece; IR, Iran; IT, Italy; JP, Japan; LB, Lebanon; LK, Sri Lanka; NI, Nicaragua; NZ, New Zealand; PK, Pakistan; SA, South Africa; SP, Spain; UK, United Kingdom.

The prevalence of disabling LBP at follow‐up varied from 6% in Japanese sales workers to 46% in Nicaraguan nurses (Fig. 1). Within individual countries, nurses tended to have more disabling LBP than office workers, the mean ratio of prevalence rates across 12 countries being 1.6 (median 1.4, inter‐quartile range: 1.1–1.7). However, the differences were smaller than those between office workers in different countries, whose prevalence ranged from 7% in Pakistan, 10% in Sri Lanka, and 11% in the UK and Japan to >30% in Ecuador, Iran, Nicaragua and Brazil. Similarly, there was nearly fourfold variation between countries in the prevalence among nurses.

Mean pain propensity indices by occupational group also varied markedly, ranging from 0.2 in Brazilian sugar cane cutters and 0.6 among office workers in Pakistan to 3.3 in manual workers in Ecuador. As illustrated in Fig. 1, there was a clear correlation across the 45 occupational groups between the prevalence of disabling LBP in the past month at follow‐up and the mean pain propensity index at baseline (Spearman correlation coefficient = 0.58).

When no account was taken of any risk factors, the dispersion of prevalence rates by occupational group was much greater than would have been expected by chance. Thus, the geometric mean of the ratio of observed to expected prevalence rates was 1.68, whereas a value less than 1.23 would have been expected at the 95% level. When account was taken of individual pain propensity, the dispersion of observed to expected ratios was reduced (geometric SD: 1.58), although still greater than the 95th centile value from randomized simulations (1.37). Adjustment also for other risk factors reduced the dispersion further (geometric SD: 1.49), such that it fell between the 75th and 95th centile of the expected distribution.

## 4 Discussion and conclusions

Our findings confirm large differences in the prevalence of disabling LBP between countries, even among workers carrying out similar occupational activities. These differences, which were substantially greater than those between nurses and office workers within the same country, appear to be driven largely by unidentified factors predisposing to musculoskeletal pain at multiple anatomical sites. After allowance for other known and suspected risk factors, including occupation, the strongest risk factor for future prevalent disabling LBP in individual participants was the number of other anatomical sites that had been painful in the year before baseline; while in occupational groups, the prevalence of disabling LBP at follow‐up correlated with the mean number of sites outside the low back that had earlier been reported as painful. This pattern of results suggests that much of the global burden of disability from LBP in working populations will not be eliminated by current ergonomic approaches to prevention which focus largely on mechanical loading of the spine, and indicates a need to understand better why workers in some countries are more prone to musculoskeletal pain in general.

Our analysis had the advantages of a large and geographically diverse study sample, with a longitudinal design and a fairly high response rate at follow‐up. The occupational groups studied were selected to allow comparison of workers carrying out similar occupational tasks in differing cultural environments, with participation restricted to men and women who initially were aged 20–59 years. Therefore, the study samples will not necessarily have been nationally representative, particularly in their exposure to occupational risk factors and their prevalence of musculoskeletal pain and disability. However, the associations of pain outcomes with risk factors can probably be generalized with greater confidence (although we included only one group of agricultural workers, whose relative risks of LBP may be exceptionally high (Driscoll et al., 2014)).

It is possible that in some occupational groups, a few potential participants were excluded because at the time of the baseline survey they were absent from work as a consequence of musculoskeletal disorders. Moreover, response rates at follow‐up were a little higher among participants who had more pain outside the low back at baseline. However, selective participation would cause serious bias only if the workers who completed follow‐up were substantially unrepresentative in the association of pain at other sites with later disabling LBP, and this seems unlikely.

Musculoskeletal pain is often persistent or recurrent, and 83% of the participants with disabling LBP at follow‐up had also suffered from LBP in the 12 months before baseline. However, we excluded LBP from our measure of pain propensity, and we have no reason to expect that earlier experience and report of pain at sites other than the low back would seriously bias report of disability from LBP at follow‐up. It might be that pain, particularly at multiple sites, lowers mood, rendering people more vulnerable to future symptoms and less able to cope with them when they occur. However, the association that we observed with pain propensity was apparent after adjustment for mental health.

Our data were collected by questionnaire, and we did not make a detailed assessment of ergonomic exposures. However, our regression analyses used random intercepts to allow for differences in the frequency of disabling LBP between occupational groups that were not explained by other risk factors in the models. Since each occupational group was selected to be fairly uniform in its occupational activities, this adjustment will have helped to account for effects of unmeasured ergonomic exposures. Furthermore, the risk factors in our final analysis accounted for most, if not all, of the variation between occupational groups in the prevalence of disabling LBP, beyond that which could be expected simply by chance. This suggests that we did not overlook any important risk factors acting independently of those in our model.

Variation between individuals in our measure of pain propensity could reflect differences either in their experience of pain, or in their inclination to report it, and since pain is an entirely subjective experience, there was no meaningful way of distinguishing between these two possibilities. Importantly, however, the outcome with which it was associated, was not report of LBP per se, but of disability for everyday activities because of LBP. There may have been some errors in recall of pain over the 12 months before baseline, but we have no reason to expect that it would be differential with respect to later report of disabling LBP at follow‐up, and any bias is therefore likely to have been towards the null.

Our reason for adopting a longitudinal design, in which risk factors were assessed at an earlier time‐point than the outcome (1‐month prevalence of disabling LBP), was that it guarded against the bias which can occur when risk factors and outcomes are assessed simultaneously. Nevertheless, it remains possible that baseline report of some risk factors was affected by the presence of disabling LBP in those participants who already had the symptom at that time.

As in earlier papers based on the CUPID study (Coggon et al., 2013a; Vargas‐Prada et al., 2013), we classed LBP as disabling if it made it difficult or impossible to get dressed, do normal jobs around the house or cut toe nails. This accords with the dictionary definition of ‘disabling’ as interfering with the way that someone can live their life, and was intended to distinguish symptoms that were more severe. The specification did not require disabling LBP to have been persistent, but 83% of the 2003 participants with disabling LBP in the past month at follow‐up had also reported LBP in the 12 months before baseline, indicating that in most cases the pain was in fact chronic or recurrent.

We took the prevalence of disabling LBP as our outcome (rather than its incidence) because the starting point for our investigation was unexplained variation in prevalence between occupational groups in different countries. The extent to which the observed associations reflected effects on the incidence of new episodes of LBP as opposed to the persistence or recurrence of pain that had already developed will be the subject of a future report. However, it is known from previous research that pain at other anatomical sites predicts the persistence of LBP (Mallen et al., 2007).

The association between LBP and earlier pain at other anatomical sites could have occurred through three mechanisms. First, pain elsewhere might make pain in the back more likely to develop or persist, perhaps through biomechanical effects of changes in posture or movement, or through altered central processing of pain. In practice any such effects are likely to be small, and we are not aware of evidence, for example, that upper limb fracture is importantly associated with LBP. Second, back pain could promote the occurrence or persistence of pain at other anatomical sites. For similar reasons, we think that is unlikely to be a major effect. Third, there could be one or more shared risk factors that predispose both to LBP and to pain at multiple other anatomical sites. This was our prior hypothesis, and seems the most likely explanation. The shared determinant(s) could be intrinsic psychological or physiological characteristics, or (currently unrecognized) external factors. Whatever their nature, our data suggest that they are important, and accounted for much of the variation in disabling LBP between our occupational groups.

Somatizing tendency is known to be strongly associated with multi‐site pain (Coggon et al., 2013b), and as expected, pain propensity was greater in participants who reported distress from common somatic symptoms (Table 2). Somatizing tendency is also a risk factor for LBP specifically (Pincus et al., 2002; Vargas‐Prada et al., 2013), but as for mental health, the association of disabling LBP with pain propensity was present after adjustment for tendency to somatize. It may be that among people who are predisposed to notice and worry about common somatic symptoms, some are particularly sensitive to musculoskeletal pain. When account was taken of both pain propensity and somatizing tendency, the PAF exceeded 50%.

The associations that we observed with other personal risk factors were largely as expected. Although the PRR for heavy lifting at work was relatively low (1.1), this may in part have been a consequence of the study design, such that there was more variation in occupational tasks between than within the occupational groups sampled. Thus, some of the effect of occupational lifting may have been obscured in the random intercept modelling that was used to allow for possible clustering by occupational group. However, while nurses tended to suffer more from disabling LBP than office workers, type of occupation accounted for less of the variation between occupational groups than mean pain propensity index.

Good ergonomics has clear benefits – it makes tasks more comfortable, and may enable people with musculoskeletal disorders to work productively when otherwise they could not. Moreover, it could be that trials to date have not tested the forms of ergonomic intervention that would be most effective in preventing LBP. However, our results reinforce the limitations of ergonomics alone as a means of preventing LBP in the workplace, and suggest that a focus also on modifying wider propensity to pain and tendency to somatize could be more productive.

As well as personal risk factors, we also explored the influence of characteristics relating to occupational groups. To reduce the possibility of spurious findings because some of the group‐level variables were mutually associated, we examined each independently, with adjustment only for personal risk factors. When analysed in this way, only one (lack of social security for long‐term unemployment) showed a statistically significant association with disabling LBP. If anything, this variable would have been expected to operate in the reverse direction, the financial threat of job loss acting as a disincentive to focusing on and worrying about pain. Thus, the association may have occurred simply by chance.

In conclusion, our analysis reaffirms wide international variation in the prevalence of disabling LBP, and indicates that, at least in the occupational groups studied, relatively little of this variation is attributable to causes specific to the low back – either physical or psychological. Rather the major driver appears to be factors that predispose to musculoskeletal pain more generally. An implication of this finding is that ergonomic interventions of the type that have been widely pursued in developed countries may have only limited impact on the global burden of disability from LBP, and that added potential for prevention may lie in understanding what determines general propensity to musculoskeletal pain, and how that propensity can be reduced to the low levels that currently occur in countries such as Pakistan, Japan and Sri Lanka.

## Author contributions

David Coggon initiated and coordinated the CUPID study, led data collection in the UK, and wrote the first draft of the manuscript; Georgia Ntani carried out the statistical analysis; Keith Palmer provided input to the design of the CUPID study, and to the interpretation of findings; Vanda Felli led data collection in Brazil; Florencia Harari led data collection in Ecuador; Leonardo A Quintana coordinated data collection in Colombia; Sarah Felknor and Marianela Rojas led data collection in Costa Rica and Nicaragua; Anna Cattrell coordinated data collection in the UK; Sergio Vargas‐Prada coordinated data collection in Spain; Matteo Bonzini led data collection in Italy; Eleni Solidaki led data collection in Greece; Eda Merisalu led data collection in Estonia; Rima Habib led data collection in Lebanon; Farideh Sadeghian led data collection in Iran; Masood Kadir led data collection in Pakistan; Sudath Warnakulasuriya coordinated data collection in Sri Lanka; Ko Matsudaira led data collection in Japan; Busisiwe Nyantumbu led data collection in South Africa; Helen L Kelsall coordinated data collection in Australia; Helen Harcombe led data collection in New Zealand. In addition, all authors provided feedback on the initial draft manuscript, and agreed the final changes.
